# THINKING OUTSIDE THE BOX: THE ROLE OF FUNDING AND COMMUNITY-ENGAGED RESEARCH IN THE FUTURE OF AREA AGENCIES ON AGING

**DOI:** 10.1093/geroni/igae098.0648

**Published:** 2024-12-31

**Authors:** Katie White

**Affiliations:** Central Ohio Area Agency on Aging, Columbus, Ohio, United States

## Abstract

As the older adult and disabled populations continue to grow, Area Agencies on Aging (AAAs) must develop new ways to meet the ever-diverse needs and ideas of individuals in their regions through innovative research partnerships. AAAs have served as the federally mandated, local leaders on aging and disability by planning, developing, funding, and implementing local systems of coordinated home and community-based services (HCBS). The Older Americans Act is the cornerstone of non-Medicaid HCBS, providing funding and requirements for nutrition, supportive, caregiver, and evidence-based services. By partnering with the Age-Friendly Innovation Center of The Ohio State University College of Social Work (AFIC), the Central Ohio Area Agency on Aging (COAAA) is supporting community-engaged participatory research with older adults and people with disabilities to inform future policy, program, service, and funding priorities. This presentation will outline how the COAAA prioritized funding community-engaged research, applied research findings to policy initiatives, and infused evidence-informed practice in new and emerging areas of focus. Practical steps for researchers to engage effectively with area agencies on aging will be discussed.

